# Serum strain-specific or cross-reactive neuraminidase inhibiting antibodies against pandemic А/California/07/2009(H1N1) influenza in healthy volunteers

**DOI:** 10.1186/s13104-015-1086-z

**Published:** 2015-04-10

**Authors:** Yulia A Desheva, Tatiana A Smolonogina, Svetlana A Donina, Larisa G Rudenko

**Affiliations:** Institute of Experimental Medicine, Russian Academy of the Medical Sciences, Acad. Pavlov’s Street, 12, Saint Petersburg, 197376 Russian Federation

**Keywords:** Influenza virus, Neuraminidase, Anti-neuraminidase antibodies, Pandemic influenza, A/H1N1

## Abstract

**Background:**

Pre-existing antibodies to influenza virus neuraminidase may provide protection against infection influenza viruses containing novel hemagglutinin (HA). The aim of our study was to evaluate serum neuraminidase-inhibiting (NI) antibodies against А/California/07/2009(H1N1) [H1N1/2009pdm] and А/New Caledonia/20/1999(H1N1) [H1N1/1999] influenza viruses in relation with the age of participants and hemagglutination-inhibition (HI) antibody levels. Anti-H1N1/2009pdm neuraminidase and anti-H1N1/1999 neuraminidase antibody levels were measured in total 219 serum samples from Russian healthy peoples of various ages examined before and a year after pandemic strain appearance. We adjusted peroxidase-linked lectin micro-procedure to measure NI antibody titers using the reassortant A/H7N1 influenza viruses based on A/equine/Prague/1/56(H7N7). Also, HI antibody titers were estimated against H1N1/2009pdm, H1N1/1999 and a panel of seasonal A/H1N1 influenza viruses.

**Results:**

In sera samples collected during the fall of 2010, mean titers of specific HI and NI antibodies to H1N1/2009pdm were 2–2.1 times lower than antibody levels against H1N1/1999. Of the 163 individuals examined, 58 (35.6%) had NI anti-H1N1/2009pdm antibody titers > 1:20, compared to 93 (57.1%) who had NI anti-H1N1/1999 antibody titers > 1:20. There were low correlations between HI and NI antibody levels against either H1N1/1999 or H1N1/2009pdm in the same serum samples. The 24 adults born between 1957 and 1977 expressed very low levels of NI antibodies to A/H1N1 influenza viruses. Persons with low HI anti-H1N1/2009pdm titers but positive to seasonal A/H1N1 demonstrated significantly higher NI anti-A/H1N1 antibody titers than unexposed subjects. In 2005 cross-reactive NI anti-H1N1/2009pdm antibody titers > 1:20 were detected among 7.1% of young people.

**Conclusions:**

Our study confirmed that contact with seasonal influenza viruses may have contributed to generating the cross-reacting anti-H1N1/2009pdm NI antibodies which were detected in the sera of 18-20 years old people examined before the pandemic virus active circulation. The lowest levels of antibodies to the neuraminidase of N1 subtype were in the group of participants born during the circulation of influenza A/H2N2 or A/H3N2 viruses. The low correlation between HI and NI antibody titers suggests that NI antibody detection can be used as an additional test to evaluate the immune response after influenza infections or immunizations.

## Background

In April 2009, the World Health Organization registered the first 21st century pandemic caused by a type А/H1N1 influenza virus (the genus *Influenzavirus,* the family *Orthomyxoviridae*) not previously isolated from animals or humans. Pandemic influenza, in contrast to seasonal influenza, affected young people more frequently than elderly [1]. In the USA, 79% of laboratory-confirmed cases of pandemic H1N1/2009pdm infections were from persons younger than 30 years, and 2% from age group older than 60 years [2]. A number of publications have analyzed pre-existing neutralizing antibodies and T-cell immunity against H1N1/2009pdm [2,3], although little is known about pre-existing cross-reactive anti-neuraminidase (NA), or neuraminidase-inhibiting (NI) antibodies to pandemic A/H1N1 in humans. Several animal studies, including a plasmid DNA vaccine model, suggest that NI antibodies could provide partial protection from lung infection and even from lethal challenge with highly pathogenic А/H5N1 influenza viruses [4-7].

In humans, NI antibodies play a role in decreasing the severity of natural infection caused by influenza A shift or drift variants [8]. Previously it was shown that immunization with seasonal influenza strains induced cross-reactive serum antibody to the NA of antigenically distinct H1N1/2009pdm, mostly in elderly individuals [9,10].

The aim of our current study was to examine the presence of homologous and cross-reactive NI antibodies against H1N1/2009pdm in serum samples collected in the fall of 2010 from healthy Russian people for more detailed estimation of the overall and age-specific influenza immunity.

## Results and discussion

**Figure 1:**
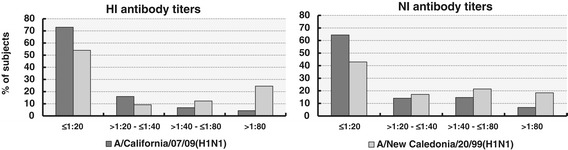
**Serum antibody titers against H1N1/1999 and H1N1/2009pdm in the fall of 2010.** Distribution of hemagglutination-inhibition and neuraminidase-inhibition antibody titers against seasonal and pandemic A/H1N1 influenza viruses in serum samples collected from 163 volunteers in the fall of 2010.

We estimated HI and NI antibodies against both H1N1/2009pdm and seasonal H1N1/1999 influenza viruses in 163 sera samples obtained in the fall of 2010 (Figure 1). Forty-four of the 163 individuals examined (27%) had HI H1N1/2009pdm antibody titers > 1:20, while 75 (46%) had HI H1N1/1999 antibody titers > 1:20. Also, 93 (57.1%) of the participants expressed NI H1N1/1999 antibodies in titers > 1:20, while only 58 (35.6%) expressed NI H1N1/2009pdm antibodies in such titers (McNemar test: p < 0.001).

From the 163 examined sera among 39.3% were detected antibody titers > 1:20 against both HI and NI of H1N1/1999; 6.7% and 17.8% expressed only HI or NI antibodies against H1N1/1999 in such titers, respectively. In comparison, 17.8% sera were double-positive to both H1N1/2009pdm surface antigens; 9.2% and 17.8% expressed only HI- or NI-antibodies against pandemic strain in titers > 1:20, respectively. Chi-square test’s p-value < 0.001 suggested a statistically significant relationship between HI and NI antibody levels against each A/H1N1 in the same serum samples, although the correlation was rather low (Spearman rs = 0.32 [95% CI: 0.11–0.57], and rs = 0.29 [95% CI: 0.01–0.54], in case of H1N1/1999 and H1N1/2009pdm respectively). Forty-eight (29.5%) of subjects had NI titers > 1:20 against both H1N1/1999 and H1N1/2009pdm [95% CI: 23.0% –36.9%].

The age distribution of A/H1N1-specific antibodies was analyzed in several age groups of participants. Persons in group 1 were born prior to 1957; group 2: 1957–1976; group 3: 1977–1999; and group 4, after 2000 (Table 1). Only group 1 expressed NI antibodies against H1N1/2009pdm in significantly higher mean titers than HI antibodies (Table 1). The highest mean titers of NI antibodies against both H1N1/2009pdm and H1N1/1999 were detected in group 3.

**Table 1:** **Titers of antibodies against A/H1N1 influenza in volunteers of different ages examined in 2010**

**Groups**	**Year of birth**	**Antibodies against H1N1/2009pdm, log2**		**Antibodies against H1N1/1999, log2**	
		**HI titers, Me (Q1;Q3)**	**NI titers, Me (Q1;Q3)**	**HI titers, Me (Q1;Q3)**	**NI titers, Me (Q1;Q3)**
1 (n = 24)	Before 1957	2.3^1^ (2.3;2.3)	3.5 (2.3;5.3)	2.3^2^ (2.3;2.8)	3.9 (2.3;5.1)
2 (n = 24)	1957–1976	2.3 (2.3;5.3)	2.3^3^ (2.3;3.3)	2.3 (2.3;4.3)	3.8 (2.3;4.6)
3 (n = 78)	1977–1999	4.3^4^ (3.3;5.3)	4.3 (3.4;5.3)	6.3^5^ (5.3;8.3)	5.7^6^ (4.8;6.4)
4 (n = 37)	After 2000	2.3 (2.3;5.3)	2.9 (2.3;5.3)	2.3 (2.3;5.3)	3.4 (2.3;5.3)

Key: Me (medians), Q1; Q3 (lower and upper quartiles).

^1^Mean titers of HI antibodies against H1N1/2009pdm are lower than NI antibodies (p = 0.001).

^2^Mean titers of HI antibodies against H1N1/1999 are lower than NI antibodies (p = 0.006).

^3^Mean titers of antibodies against NA of pandemic influenza virus H1N1/2009pdm in are lower in group 2 than in group 3 (p = 0.002).

^4^Mean titers of HI antibodies against H1N1/2009pdm are higher in group 3 than in group 1 (p = 0.0002).

^5^Mean titers of HI antibodies against H1N1/1999 are higher in group 3 than in groups 1, 2, and 4 (p < 0.0001).

^6^Mean titers of NI antibodies H1N1/1999 are higher in group 3 than in groups 1, 2, and 4 (p < 0.001).

**Figure 2:**
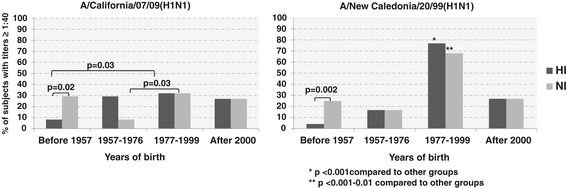
**Hemagglutination-inhibition and neuraminidase-inhibition antibodies seroprotection rates in dependence of birth date.** Seroprotection rates were defined as a percentage of subjects with titers ≥ 1:40 of hemagglutination-inhibition and neuraminidase-inhibition antibodies against H1N1/1999 or H1N1/2009pdm influenza viruses among 163 individuals examined in the fall of 2010.

Figure 2 presents the seroprotection rates (proportions of subjects with antibody titers ≥ 1:40) [11] among groups of participants. Again participants in group 3 (1977–1999 years of birth) had the levels of herd immunity to both HA and NA of seasonal H1N1/1999 even higher than children born after 2000 (group 4), when А/New Caledonia/20/99(H1N1) became dominant (Fisher’s test: p = 0.0001; see Figure 2). This phenomenon could be attributed to higher social activity of older people from group 3 over young children, leading to more infections and development of cross-reactive antibodies to previously circulating A/H1N1 influenza viruses.

Participants born between 1957 and 1976 possessed the lowest levels of NI antibodies against both H1N1/1999 and H1N1/2009pdm influenza viruses (Table 1, Figure 2).

To determine a possible origin of NI antibodies against H1N1/2009pdm, we evaluated antibody titers ≥1:40 supposed to be protective in three groups separated on the basis of pre-existing HI antibody levels against H1N1/2009pdm and seasonal A/H1N1 viruses (n = 159, 4 sera were excluded from analysis because there was no HI data with all tested A/H1N1 antigens). Group I were persons with low HI titers (≤ 1:10) against H1N1/2009pdm and no antibodies to other A/H1N1 viruses (≤ 1:20); group II were persons with low HI titers (≤ 1:10) against H1N1/2009pdm and positive (HI titers ≥ 1:40) for any of the other tested A/H1N1 influenza viruses (see Methods); and group III included all persons with HI titers against H1N1/2009pdm ≥ 1:20 (Table 2). Group I subjects, who were negative to all A/H1N1 viruses used in the HI test, had a higher median age (45 years) than group II (17 years) and group III (15 years), and a wider variation of age (Kolmogorov-Smirnov’s test: p < 0.005; see Table 2).

**Table 2:** **Homologous and cross-reactive antibodies against NA of H1N1/2009pdm among participants examined in 2010**

**Group**	**HI antibody levels against A/H1N1 influenza viruses**		**Age, years Me(Q1;Q3)**	**Antibodies to H1N1/2009pdm**				**Antibodies to H1N1/1999**			
				**HI titers, log2**		**NI titers, log2**		**HI titers, log2**		**NI titers, log2**	
	**H1N1/2009pdm**	**seasonal A/H1N1 viruses**		**Me (Q1;Q3)**	**% ≥ 1:40**	**Me (Q1;Q3)**	**% ≥ 1:40**	**Me (Q1;Q3)**	**% ≥ 1:40**	**Me (Q1;Q3)**	**% ≥ 1:40**
I	≤1:10	≤ 1:20	45 (6; 58)	2.3 (2.3;2.3)	0	2.3 (2.3;3.2)	2.4	2.3(2.3; 2.3)	0	2.3 (2.3; 4.1)	2.4
N = 41											
II	≤1:10	≥ 1:40	17 (10; 26)	2.3^1^ (2.3; 3.3)	0	3.4^2^ (2.3; 4.3)	15.7	6.2(4.3; 8.3)	72.5	5.3 (4.1; 6.2)	51.0^3^
N = 51											
III	≥ 1:20	any	15 (12; 20)	5.3 (4.3; 6.3)	65.7	5.2^4^ (3.9; 5.6)	38.8	5.3(2.3; 7.3)	56.7	5.5 (3.9; 6.4)	55.2
N = 67											

Key: N (number), Me (medians), Q1; Q3 (lower and upper quartiles); % (percent).

^1^Titers of HI antibodies against H1N1/2009pdm are similar in group I and group II (Dunn’s test: z’ = 0.959, p = 1.0).

^2^Titers of NI antibodies H1N1/2009pdm are higher in group II than in group I (Dunn’s test: z′ = 2.421, p = 0.046).

^3^In group II, percentage of volunteers with antibody titers ≥ 1:40 to NA of seasonal H1N1/1999 higher than to NA of H1N1/2009pdm (McNemar test: p < 0.001).

^4^Titers of NI antibodies against H1N1/2009pdm are higher in group III than in group II (Dunn’s test: z′ = 4.043, p = 0.0002).

Persons seropositive to seasonal A/H1N1 viruses but with low HI titers against H1N1/2009pdm (group II) had significantly higher NI titers against H1N1/2009pdm than unexposed subjects from group I (p < 0.05). Two adults from group II, aged 64 and 56 years, demonstrated 1:40 HI titers against А/Khabarovsk/1/77(H1N1) seasonal influenza virus and NI antibodies against H1N1/2009pdm in titers of 1:696 and 1:180, respectively. These data suggest that exposure to seasonal A/H1N1 viruses may induce cross-reactive NI antibodies against H1N1/2009pdm in high titers. Nevertheless, group II contained significantly fewer persons with NI antibody titers ≥ 1:40 against H1N1/2009pdm than against seasonal H1N1/1999 (McNemar test: p < 0.001).

**Figure 3:**
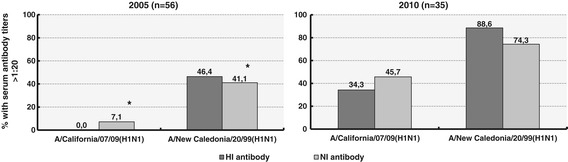
**Serum antibodies against A/H1N1 influenza viruses during 2005 and 2010 flu seasons.** Percentage of seropositive 18–20-year-old persons with titers > 1:20 of hemagglutination-inhibition and neuraminidase-inhibition antibodies against seasonal and pandemic A/H1N1 influenza viruses; * indicates p < 0.001, McNemar test.

To reveal true cross-reactive NI antibodies against H1N1/2009pdm, we also investigated levels of antibodies against H1N1/2009pdm in serum samples collected from 18–20-year-old people in the fall of 2005, long before H1N1/2009pdm arose (Figure 3). None of these subjects had detectable anti-H1N1/2009pdm HI antibodies. Only 7.1% (4 of 56) had NI antibody titers > 1:20 against H1N1/2009pdm compared to 41.1% (23 of 56) with NI antibody titers > 1:20 against epidemic H1N1/1999 (McNemar test: p < 0.001). In contrast, 34.3% (12 of 35) of samples collected from young adults of the same age in 2010 contained HI antibodies in titers > 1:20 against H1N1/2009pdm, and 45.7% (16 of 35) participants demonstrated NI antibodies to pandemic virus in titers > 1:20.

A year after the pandemic A/H1N1 virus first emerged in Russian population the mean titers of specific HI and NI antibodies against H1N1/2009pdm in all examined volunteers were 2–2.1 times lower than levels of antibodies against H1N1/1999, which circulated in Russia 1999–2006. Likewise Cramer *et al.* [12] previously reported that in 2010 the threshold level of herd immunity against H1N1/2009pdm was not achieved in Hamburg, Germany.

In our study, the participants born between 1977 and 1999 were the most likely to be infected to pandemic H1N1/2009pdm compared to those born before 1957. This finding confirmed the extensive published data about the high frequency of H1N1/2009pdm infection in children, teenagers, and adults younger than 35 years during the 2009 pandemic, varying between 20–60% in different countries [13]. High anti-H1N1/2009pdm NA antibody levels, rather than anti-HA antibody levels, in participants older than 53 years can be attributed to contact with earlier circulating A/H1N1 viruses. Examining ages of persons with no antibodies against H1N1/2009pdm HA (HI titers ≤ 1:10), we found anti-H1N1/2009pdm NA antibodies in titers ≥ 1:40 in 22.7% (5 of 22), 12.9% (4 of 31), and 3.8% (1 of 26) of participants born before 1957, in 1977–1999, and after 2000, respectively, but none in persons born between 1957 and 1977. Thus, in our study differences in levels of anti-H1N1/2009pdm NA antibodies in people without direct contact with pandemic virus depended on age and, possibly, on priming by previous epidemic influenza viruses. Indeed, volunteers born in 1957–1976, when influenza viruses A/H2N2 and A/H3N2 were circulating, were the least likely to have anti-H1N1/2009pdm NA antibody titers ≥ 1:40, but about one third of participants from this age group had anti-H1N1/2009pdm HI antibody titers ≥ 1:40 (Figure 2), most likely due to pandemic virus natural infection.

The low correlation between HI and NI antibody titers we found can be attributed to the autonomy of the serum immune response to both surface glycoproteins of the influenza virus, widely reviewed in scientific literature [14-16]. Other reasons for such divergence may be different times of persistence of NI and HI antibodies, according to individual anamnesis of the surveyed influenza infections [17,18], and the ability of anti-NA antibodies to interact with a wider spectrum of viruses than anti-HI antibodies. Thus, some persons with no antibodies against HA of influenza pandemic virus in the fall of 2010 still may have been exposed to H1N1/2009pdm. Nevertheless, high levels of antibodies against H1N1/2009pdm NA among participants not expressing anti-H1N1/2009pdm HA of pandemic virus, but positive to epidemic H1N1 viruses HA, compared with completely negative individuals examined during the same epidemic period, may, to some extent, confirm the relationship between exposure to epidemic A/H1N1 viruses and development of antibodies cross-reactive with H1N1/2009pdm NA. However, the most reliable data about truly cross-reactive antibodies to H1N1/2009pdm may be obtained using sera collected long before the appearance of pandemic virus. During the 2005 epidemic season, the 7.1% of the 56 examined in our study 18–20-year-old volunteers had anti-pandemic virus NI antibodies in titers > 1:20.

The most convincing data concerning the protective action of pre-existing anti-NA antibodies were obtained in the 1970s using a large cohort of volunteers with no or low levels of anti-HA antibodies against pandemic influenza. One such study, by Monto *et al.* [8], showed that, prior to the Hong Kong A/H3N2 influenza pandemic, only 12% of the population had high NI antibody titers to NA of N2 subtype, while 72% had none. The relatively low level of herd immunity to A/H2N2 NA in the population correlated with the wide distribution of the new pandemic subtype A/H3N2 virus in 1968. However, this herd immunity was nonetheless sufficient to moderate the severity of the pandemic: the frequency of confirmed A/H3N2 influenza infection, determined by elevated levels of HI antibodies, was reversely proportional to pre-existing levels of anti-N2 antibodies. The possible effect of HA was eliminated because the sera were obtained before the virus with the novel H3 HA had appeared. Volunteers between 20 and 45 years of age who had anti-N2 antibodies in titers ≥ 1:16 were 2.0–2.6-fold less likely to develop respiratory infection symptoms than persons with low NI antibody titers. The authors suggested that neuraminidase antibody can protect not so much against infection as against symptoms of influenza thus permitting the individual to ‘up-date’ his antibody status from time to time without suffering clinical influenza [8]. The levels of protective NI antibodies still unclear although protective HI antibody titers defined as ≥ 1:40 [11]. The results of several studies suggest the different levels of NI antibodies obtained in several laboratories (1:8- ≥ 1:20) may be protective against natural influenza infection [8,18,19].

## Conclusions

Our study confirmed that contact with seasonal influenza viruses may have contributed to generating the cross-reacting antibodies against NA of H1N1/2009pdm. Indeed, сross-reactive anti-H1N1/2009pdm NA present in the sera of individuals negative to pandemic virus HA, but positive to epidemic strains of A/H1N1 subtype. NI antibodies against the pandemic virus were detected among 7.1% of volunteers 18-20 years old examined in 2005, several years before this virus actually broke out. The lowest levels of antibodies to the NA of N1 subtype belonging to either H1N1/2009pdm or H1N1/1999 were in the group of participants 1957-1977 years of birth, i.e. born during the circulation of influenza A/H2N2 or A/H3N2 viruses.

The low correlation between HI and NI antibody titers we found suggests that NI antibody detection can be used as an additional test to evaluate the immune response after influenza infections or immunizations.

## Methods

### Viruses

The A/H7N1 reassortant influenza virus containing A/California/07/2009(H1N1) NA and A/equine/Prague/1/56(H7N7) HA was generated by classical genetic reassortment in embryonated chicken eggs [20]. Parental A/equine/Prague/1/56(H7N7) influenza virus was kindly provided by Dr. Klimov at the CDC (Atlanta, GA, USA). The other A/H7N1 reassortant influenza virus containing А/New Caledonia/20/1999(H1N1) NA and A/equine/Prague/1/56(H7N7) HA was provided by the Institute of Influenza, Ministry of Healthcare of the Russian Federation, Saint Petersburg, Russian Federation.

### Serum samples

163 sera were collected in the fall of 2010 from Russian people aged 2–83 years. Patient history regarding previous influenza infections or vaccinations was unknown. These serum samples left as part of routine tests. We also tested 56 sera left as part of screening tests of young adults examined before vaccination with seasonal influenza vaccine in the fall of 2005.

### Ethics statement

In our retrospective study we used only serum samples left as a part of routine tests. These serum samples were provided by the Diagnostics Laboratories (Saint Petersburg, Russian Federation).

### Serum antibody evaluation

Sera were treated with receptor-destroying enzyme from *Vibrio cholerae* (Denka-Seiken, Tokyo, Japan), and tested in duplicate for hemagglutination-inhibition H1-specific antibodies using standard procedures [21] with the following test antigens: live influenza vaccine viruses А/17/California/09/38(H1N1), А/17/New Caledonia/99/145(H1N1), А/17/Solomon Islands/06/9(H1N1), А/17/Brisbane/07/28(H1N1); seasonal influenza virus 1977 year of isolation А/Khabarovsk/1/77(H1N1) and А/Puerto Rico/8/34(H1N1).

The peroxidase-linked lectin micro-procedure previously reported by Lambré *et al*. [22] was adjusted to assay NI antibodies using diagnostic A/H7N1 reassortant viruses. Serum samples were heated at 56°C for 30 min, serially diluted in PBS-BSA with the pH = 6.9 (typically, seven 2-fold dilutions starting at 1:10). Sixty μL of serum dilutions were incubated with an equal volume of pre-diluted virus containing 128 HA units for 30 min at 37°C. After incubation, 100 μL of the mixture was added to the 96-well plates (Sarstedt AG & Co, Nümbrecht, Germany) coated with 150 μL of 50 μg/mL fetuin (Sigma-Aldrich, St. Louis, MO, USA). After 2 hours incubation at 37°C the plates were washed, and NA activity was measured by incubating with peroxidase-labeled peanut lectin (2.5 μg/mL; Sigma-Aldrich, St. Louis, MO, USA) for 1 h at room temperature, washing, and adding 100 μL of peroxidase substrate. The reaction was stopped after 5 min with 100 μL of 1 N sulfuric acid. OD values were measured at 450 nm using the universal microplate reader (ELx800, Bio-Tek Instruments Inc, Winooski, VT, USA). NI titers were expressed as the inverse of the dilution that gave 50% OD_450_ of positive control (virus without serum).

### Statistical analysis

Data were analyzed with Statistica software, version 6.0 (StatSoft, Inc. Tulsa, Oklahoma, USA). Medians (Me) and lower and upper quartiles (Q1; Q3) were calculated and used to represent the antibody response. Comparisons of two independent groups were made with nonparametric test, namely the Kolmogorov-Smirnov 2-sample test. To compare multiple independent groups, we used a Kruskal-Wallis analysis of variance (ANOVA) with subsequent multiple pairwise comparisons based on Kruskal-Wallis sums of ranks. Comparisons of two dependent variables were performed using Wilcoxon matched pairs test. In the case of nominal variables, Chi-square tests, Fisher exact 2-tailed tests, or McNemar’s chi-square tests were done. Non-parametric measure of statistical dependence between 2 variables was done using Spearman’s rank correlation coefficient. The p-value ≤ 0.05 was considered to be statistically significant. Additionally, we used the Bonferroni correction for significance levels when testing several hypotheses on a single set of data.

## Abbreviations

HA: Hemagglutinin
NA: Neuraminidase
HI: Hemagglutination inhibition
NI: Neuraminidase inhibition
Me: Medians
GMT: Geometric mean titers
